# The Current Status and Prevention of Antibiotic Pollution in Groundwater in China

**DOI:** 10.3390/ijerph191811256

**Published:** 2022-09-07

**Authors:** Huiping Zeng, Jianxue Li, Weihua Zhao, Jiaxin Xu, He Xu, Dong Li, Jie Zhang

**Affiliations:** 1 Key Laboratory of Water Quality Science and Water Environment Recovery Engineering, Beijing University of Technology, Beijing 100124, China; 2 State Key Laboratory of Urban Water Resource and Environment, Harbin Institute of Technology, Harbin 150090, China

**Keywords:** groundwater, antibiotics, occurrence

## Abstract

The problem of environmental pollution caused by the abuse of antibiotics has received increasing attention. However, only in recent years have antibiotic pollution and its risk assessment to the environment been deeply studied. Although there has been a large number of reports about the input, occurrence, destination, and influence of antibiotics in the past 10 years, systemic knowledge of antibiotics in the groundwater environment is still lacking. This review systematically expounds the sources, migration and transformation, pollution status, and potential risks to the ecological environment of antibiotics in groundwater systems, by integrating 10 years of existing research results. The results showed that 47 kinds of antibiotics in four categories, mainly sulfonamides and fluoroquinolones, have been detected; antibiotics in groundwater species will induce the production of resistance genes and cause ecological harm. In view of the entire process of antibiotics entering groundwater, the current antibiotic control methods at various levels are listed, including the control of the discharge of antibiotics at source, the removal of antibiotics in water treatment plants, and the treatment of existing antibiotic contamination in groundwater. Additionally, the future research direction of antibiotics in groundwater is pointed out, and suggestions and prospects for antibiotic control are put forward.

## 1. Introduction

Antibiotics are a class of secondary metabolites with antipathogen or other activities produced by microorganisms (including bacteria, molds, actinomycetes, etc.) in the process of life, or synthetic chemicals used to antagonize a specific pathogenic microorganism. Due to selectively inhibiting or affecting biological functions, antibiotics have been widely used in the fields of medicine, agricultural production, and livestock farming and aquaculture, mainly including tetracyclines (TCs), macrolides (MLs), sulfonamides (SAs), chloramphenicols (CPs), and fluoroquinolones (FQs) [1]. The half-life of most antibiotics is not long [2], but due to abuse and insufficient treatment, a large number of antibiotics enter the water environment every year, resulting in the phenomenon of “false persistence”. SAs are generally detected in the groundwater in most parts of the United States, and the peak concentrations of sulfamerazine (SM1) and sulfamethazine (SMZ) are as high as 360 ng/L and 1100 nm/L [3], respectively. In addition, antibiotics such as SAs, MLs, and FQs were detected in groundwater near livestock farms [4]. The peak concentrations of SAs and MLs in groundwater in Barcelona, Spain, reached 37.1 ng/L and 2980 ng/L [5], respectively. SAs were generally detected in groundwater near a large-scale farm in Germany [6]. Antibiotics in the water environment will lead to the imbalance of microbial populations, and even induce the generation of drug resistance genes, and, at the same time, they will enter the human body with the accumulation in the food chain, threatening human health [7,8,9,10].

According to statistics, the annual consumption of antibiotics in the world can reach 100,000 t~200,000 t [1]. As a country with a large population and agriculture, China ranks first in the world in both the production and use of antibiotics. In 2013, the total use of antibiotics in China reached 92,700 t, of which about 54,000 t entered the sewage treatment plant. Since they cannot be completely removed during the sewage treatment process, antibiotics eventually enter the water environment [11]. In recent years, antibiotics have been detected in major surface water systems in China, including the Pearl River Basin, the Liaohe River Basin, and the middle and lower reaches of the Yangtze River; groundwater is also affected by the interaction between surface water and groundwater, causing antibiotic pollution [12]. Per capita freshwater resources are relatively scarce in China, and groundwater is used as domestic water in some cities and most rural areas. Although in low levels, antibiotics in groundwater still pose a great threat to human health through the food chain [13,14]. However, compared to monitoring studies on organic and inorganic indicators, antibiotic contamination in groundwater has received relatively little attention.

**Figure 1:**
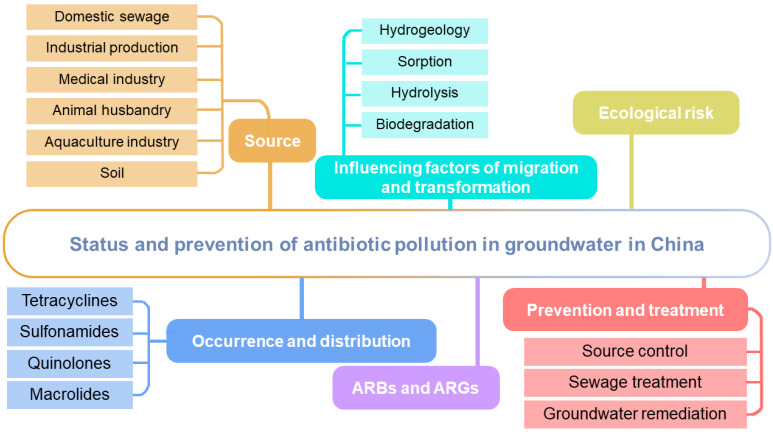
Overview of the framework.

In order to understand the occurrence status of antibiotics in the groundwater of Chinese water systems, this paper reviews the relevant literature in the past 10 years, and identifies the source, migration, and harm of antibiotics in groundwater in China, so as to provide a reference for the prevention and control of antibiotic pollution and the establishment of relevant laws and regulations. The review framework of this paper is shown in Figure 1.

## 2. Methodology

Mass production and widespread use are the main reasons for the long-term existence of antibiotics in groundwater. Antibiotics form a variety of metabolites after continuous migration and transformation in the groundwater environment, posing potential threats to the ecological environment. Therefore, the qualitative and quantitative analysis of antibiotics and metabolites in groundwater is becoming more and more important. After decades of development, the detection of antibiotics has developed from a simple observational method to an accurate qualitative and quantitative method, using sophisticated instruments. The commonly used methods at this stage are [15]: chromatography, mass spectrometry, Enzyme-Linked Immunosorbent Assay (ELISA), capillary electrophoresis, microbiology, Nuclear Magnetic Resonance Spectroscopy (NMR), and their combination methods, such as Gas Chromatography–Mass Spectrometry (GC–MS), Liquid Chromatography–Mass Spectrometry (LC–MS), quadrupole-time-of-flight tandem mass spectrometry (Q-TOF), HPLC–NMR, and high-performance liquid chromatography–tandem triple quadrupole Rod mass spectrometry (UPLC–MS/MS).

## 3. Source

**Figure 2:**
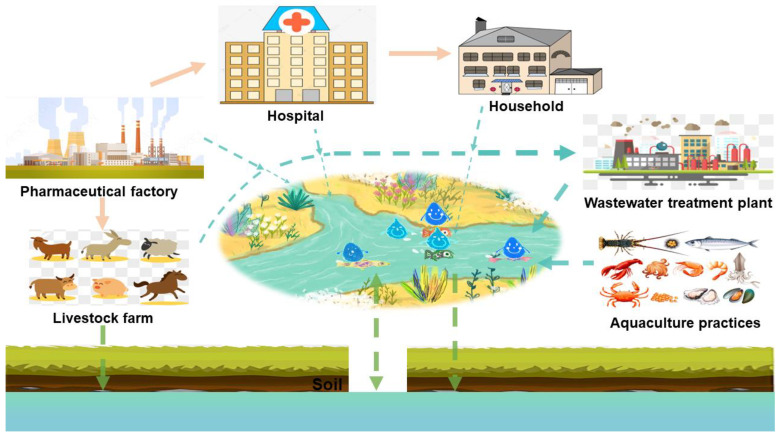
Sources of antibiotics in groundwater.

Antibiotics in the surface water and soil directly affect the groundwater environment. Among them, the sources of antibiotics in surface water mainly include domestic sewage, the medical and health industry, the antibiotic production industry, and livestock farm and aquaculture wastewater; the main sources of antibiotics in the soil environment are the use of poultry and livestock manure and landfill disposal. After the antibiotics in the surface water and soil enter the vadose zone, they eventually migrate to the groundwater through a series of complex physicochemical reactions and biological actions, such as leaching, infiltration, and groundwater–surface water interactions [3]. This article reviews six sources of antibiotics in groundwater (Figure 2).

### 3.1. Domestic Wastewater

Domestic sewage is one of the main sources of antibiotics in the environment [16]. Sewage treatment plants are the final receptors of antibiotics in urban areas, and about 50% of the antibiotics enter the water environment with the effluent of sewage treatment plants [2]. Studies have reported that antibiotics were detected in the influent and effluent of urban sewage treatment plants. Zhang et al. [17] detected 29 antibiotics in 12 sewage treatment plants in Dalian, and the cumulative concentration was 63.6 ng/L~5404.6 ng/L; FQs, SAs, TCs, and MLs accounted for 42.2%, 23.9%, 16.0%, and 14.8% of the total antibiotic concentration, respectively. Lin et al. [18] studied the effluent of a sewage treatment plant in Taiwan and found high concentrations of antibiotics, MLs, β-lactams (BLs), and imidazoles, were mostly not eliminated, and the concentrations in the effluent were even higher than those in the influent. The results of China’s 7th national census [19] showed that rural areas account for about 94% of the land, and rural domestic sewage is also one of the main sources of antibiotics in the water environment. Due to the lack of sewage collection and treatment facilities, wastewater discharged without effective treatment will cause potential ecology and health risks to the water environment [20]. Jiang et al. [21] detected a total of 55 pharmaceuticals and personal care products (PPCPs) in the Tiger Canal watershed, with a cumulative concentration of up to 647 ng/L.

### 3.2. Industrial Production

As a producer of antibiotics, the industrial wastewater from antibiotic manufacturing plants and some chemical plants contains pollutants such as raw materials, precursors, and noumenon and their metabolites, which are the first source of antibiotic pollutants in the environment. Compared with domestic sewage, the residual antibiotic concentration in antibiotic production wastewater is much higher [22], and it is difficult to remove by simple treatment technology. Li et al. [23] detected penicillin G at concentrations as high as 153 μg/L in wastewater from a pharmaceutical factory. Undegraded antibiotics and their metabolites are discharged into surface water and soil with the treated wastewater, and then enriched by leaching into groundwater. At the same time, the solid waste generated in industrial production is generally landfilled, and the antibiotic pollutants contained in it will directly enter the soil with landfill leachate or landfill drainage, and then transfer to groundwater.

### 3.3. Medical Wastewater

About 50% of hospital outpatients in China used antibiotics to treat their diseases, of which about 74% received one antibiotic and 25.3% received two or more antibiotics. Overuse of antibiotics has become a common phenomenon, especially in poor areas [24]. The excrement and discarded expired antibiotics of patients who eat and use antibiotics, as well as antibiotic residues on medical equipment, are the main sources of contamination of medical antibiotics. Wang et al. [25] detected the concentrations of 14 antibiotics in medical wastewater, and the results showed that six reached the μg/L level, which was higher than the level of antibiotics in other water environments, especially FQs, Cefalexin, and TCs. Although most urban hospitals are equipped with sewage treatment systems, their removal processes cannot effectively remove high concentrations of antibiotics, and trace amounts of antibiotics still exist in medical wastewater and enter the urban sewage pipeline system. At the same time, due to the irregular collection and storage of medical wastewater and waste in most hospitals, much medical wastewater and solid waste has already caused certain pollution to the soil and groundwater around the hospital before entering the urban solid and liquid waste treatment system.

### 3.4. Livestock Farms

Antibiotics have been widely used to prevent or treat human and animal diseases and are also used in livestock farms as growth promoters. Due to the difficulty in being absorbed by the intestine, most antibiotics are excreted, causing a health crisis [26]. It has been reported that unused antibiotics in animal waste can be transferred to surface water, groundwater, and soil, and even to aquatic and terrestrial organisms [27]. Therefore, the overuse and abuse of antibiotics in livestock farms may pollute the environment and cause antibiotic resistance [28,29]. Zhou et al. [30] detected a large amount of SAs in a farm in Guangxi Province, with the highest concentration of 128 ng/L (sulfamonomethoxine, SMM). He et al. [31] found 18 antibiotics in swine manure wastewater, including 8 SAs and diaminopyrimidines, 5 TCs, 3 MLs, and 1 lincosamide. Li et al. [32] studied the contamination of antibiotics in nine pig farms and nearby groundwater environments in North China (TCs, FQs, SAs, MLs, and fenicols), and found that antibiotics are widely distributed from wastewater to underground environments. Animal manure is an economical and readily available fertilizer for crops that has been widely used in farmland, and the antibiotics it contains will pollute the soil. Then, the contaminated soil will affect the groundwater system after the infiltration of rainwater and surface runoff [33].

### 3.5. Aquaculture Industry

With the rapid growth of the population, the demand for aquatic products is increasing, resulting in the continuous expansion of the aquaculture industry [34]. To increase the yield of aquatic products and prevent diseases, a large amount of antibiotics is often added during the feeding process. However, only a small fraction of the antibiotics is utilized, and most are discharged into the aquatic environment or sediments. Zou et al. [35] found five antibiotics at concentrations as high as 315 ng/L—mainly oxytetracycline (OTC) compounds—in the wastewater of fishponds near Bohai Bay, China. Zheng et al. [36] found higher concentrations of erythromycin (ERY), sulfamethoxazole (SMZ), and sulfamethazine (SM2) near aquaculture activities in the Beibu Gulf of China, suggesting that aquaculture can cause the pollution of antibiotics in the environment. Liang et al. [37] detected FQs, TCs, and MLs in the water and sediments of typical aquaculture areas in the Pearl River Estuary, and the average mass concentrations of the antibiotics in the water and sediments ranged from 7.63 ng/L~59.00 ng/L and 0.97 ng/L~85.25 ng/L, respectively.

### 3.6. Antibiotics in Soil

Reclaimed water irrigation, landfills, and livestock feces will introduce antibiotics into the soil, and the concentration of antibiotics in the soil is related to its source, with the highest concentrations of antibiotics in the soil near a feedlot. Zhou et al. [38] detected 12.9 mg/kg of chlortetracycline (CTC) in the soil near the discharge outlet of a pig farm. Ji et al. [39] found 4.24 mg/kg of OTC in farmland near a pig farm. Organic manures are often preferred over chemical fertilizers in the cultivation of organic vegetables. Studies have shown that the content of FQs in a vegetable growing area in Shandong, China, is relatively high, with the highest concentrations of ciprofloxacin (CIP) and ofloxacin (OFL) reaching 0.652 mg/kg and 0.288 mg/kg, respectively [40,41]. Landfill is the final disposal site for municipal solid waste, and a large amount of antibiotics will enter the landfill with domestic waste [42]. Wang et al. [43] detected 15 antibiotics in three landfills, and the total content was as high as 157.22 μg/kg~1752.01 μg/kg, of which the concentrations of FQs, TCs, Sas, MLs, and BLs were 46.02 μg/kg~1338.42 μg/kg, 5.07 μg/kg~407.87 μg/kg, 1.60 μg/kg~61.81 μg/kg, 1.96~62.91 μg/kg, and 0~99.56 μg/kg, respectively. After antibiotics are recharged into the soil environment through reclaimed water, there is a risk of downward migration and contamination of groundwater [44]. Ma et al. [45] looked at TCs, Sas, FQs, and MLs and found that the infused groundwater had higher concentrations than other groundwaters. Fang et al. [46] and Li et al. [47] also found the accumulation of antibiotics in soils recharged with reclaimed water.

## 4. Occurrence and Spatial Distribution of Antibiotics in Groundwater of China

Groundwater is the final destination of antibiotics in the environment, and its pollution levels and sources have received extensive attention at home and abroad, but systematic investigations did not begin until the end of the 20th century [48]. According to existing reports, it is found that the degree of antibiotic contamination in groundwater varies in different countries and regions, and TCs, SAs, FQs, and MLs are considered to be common antibiotic pollutants in groundwater.

Because groundwater is harder to sample than surface water, and antibiotics are difficult to detect [49], the current research based on antibiotic pollution in groundwater (9.38%) shows the percentage is less than that in surface water (90.63%) in China. After decades of development, the detection method for antibiotics in groundwater has developed from a simple observation method to an accurate and high-precision qualitative and quantitative detection method using precise instruments. Although on the one hand, antibiotics in the groundwater environment are characterized by a wide variety, low concentrations, and complex migration and transformation pathways, which require a high demand for the sensitivity, detection limit, separation effect, and qualitative and quantitative analysis of the analytical and detection instruments, on the other hand, the identification and screening of antibiotics in groundwater relies too much on the number of target antibiotics detected quantitatively, and the sensitivity and accuracy of the widely used qualitative detection instruments greatly limit the development and popularization of qualitative methods.

**Figure 3:**
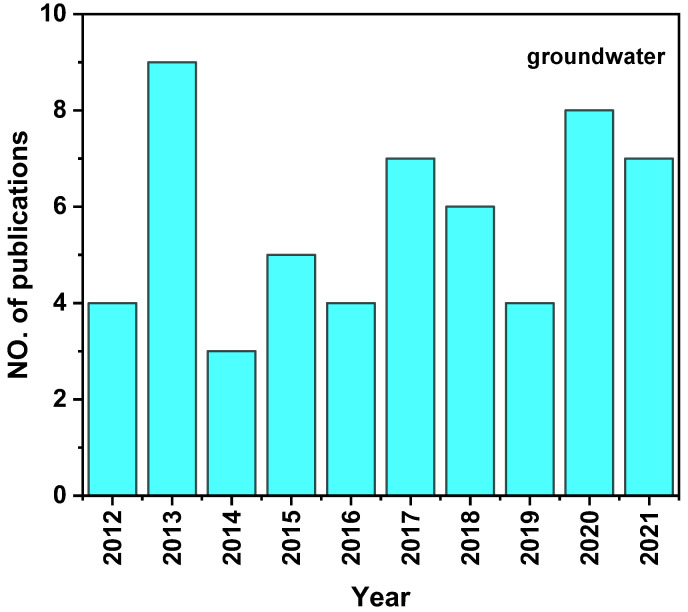
No. of publications on antibiotics in groundwater of China in previous ten years.

Based on reports of antibiotics in the groundwater in China in the past 10 years, this paper reviews the discovery and distribution of TCs, SAs, FQs, and MLs (Figure 3). The areas include Beijing, Harbin, Guilin, Taipei, Shijiazhuang, Xiong’an, Changzhou, Guangxi, Xiantao, Chengdu, Shanghai, Bijie, Shahu County, Four Lakes Basin, Chenhu Lake, the northern part of the North China Plain, North China, and Southwest China. The concentration is the maximum value, or the average value if there is no maximum value.

### 4.1. Tetracyclines

Due to its larger population and larger area of arable land, China is a major producer and user of antibiotics [50]. The detection results of TCs are shown in Table 1. In the groundwater environment of major cities in China, a total of four kinds of TCs were detected, of which the cumulative concentration of tetracycline (TC) was the highest (up to 447.11 ng/L). The highest concentration of TCs was detected in the groundwater of Shahu County in Jianghan Plain (605.51 ng/L) [51].

### 4.2. Sulfonamides

SAs are the longest-serving synthetic antibiotics in history [59], and are one of the most commonly used antibiotics in agriculture and livestock farms in recent years, and their residues in the environment and their migration and transformation have become an academic focus [60]. With the development of society, the abuse of SAs in aquaculture and agriculture has exacerbated the pollution of residual antibiotics in the environment. As shown in Table 2, no less than 19 SAs were detected in the groundwater in China. The types and concentrations of the SAs detected in different cities were different, but the cumulative concentrations of SMZ, SM2, sulfathiazole (STZ), sulfamethoxazole (SMX), and sulfachloropyridazine (SCP) were the highest, which were 394.3413 ng/L and 292.04 ng/L, 664.71 ng/L, 1967.71 ng/L and 285.55 ng/L, respectively. The cumulative concentration of SAs detected in groundwater in Taipei was the highest (1867.6 ng/L), and only the concentration of SMX was as high as 1820 ng/L [61]. However, the concentrations of SAs in Xiantao and Bijie were only 21.06 ng/L and 4.48 ng/L [51,55]. The concentration of SMZ in China’s groundwater is higher than that of South Korea (0 ng/L) [62].

### 4.3. Fluoroquinolones

FQs are one of the newest antibiotics for clinical use and are widely used in the treatment of human and animal diseases [67]. In the past decade, at least 18 FQs have been reported in the groundwater of China (Table 3). Among them, the cumulative concentrations of norfloxacin (NOR), OFL, and CIP were the highest, which were 982.78 ng/L, 1300.25 ng/L, and 324.73 ng/L, respectively. The highest concentration of FQs (896.027 ng/L) was found in groundwater in Shahu County, which was influenced by agriculture and aquaculture [51]. The concentration of FQs in the groundwater of China is higher than that of Korea (0~1.27 ng/L for NOR, 0~0.70 ng/L for OFL, 0~0.52 ng/L for CIP, 0~0.19 ng/L for ENR) [62] and the Netherlands (0 ng/L for CIP) [68].

### 4.4. Macrolides

MLs are widely used in the clinical treatment of respiratory and skin infections due to the advantages of strong penetrating power, shortening the course of disease, and a significant bacteriostatic effect [72]. As shown in Table 4, at least six MLs were detected in the reports on antibiotics in the groundwater of China in the past decade; the cumulative concentration of ERY was as high as 375.91 ng/L, and the concentration detected in Shahu County alone was 290.81 ng/L [51], followed by roxithromycin (ROX); its cumulative concentration was 61.12 ng/L.

## 5. Factors Affecting the Migration and Transformation of Antibiotics in the Groundwater System

Antibiotics have been detected in underground environments such as soil and groundwater. It has been reported that the main processes involved in antibiotics entering groundwater include sorption, hydrolysis, and microbial degradation, and that they are affected by various environmental factors and physical and chemical properties [59]. Although the purification effect of the soil layer has reduced antibiotics, some antibiotics still reach the groundwater [73]. The migration and transformation laws of antibiotics are different due to their own physicochemical properties, the soil medium, and the chemical composition of the groundwater.

### 5.1. Hydrogeology

The content of antibiotics in groundwater is related to the hydrological source and the geological characteristics of the aquifer [74]. Antibiotic-contaminated water in rivers can also pose a threat to nearby wells, and rainfall can cause pollutants such as fertilizers to enter vadose zones and aquifers. Antibiotics accumulate in saturated zones and gradually reach groundwater by gravity after precipitation [59,75].

### 5.2. Sorption

The migration of antibiotics in groundwater mainly depends on the sorption to soil and sediment. Generally speaking, antibiotics with strong sorption capacity do not easily migrate in the environment, are relatively stable, and easily accumulate, while antibiotics with weak sorption capacity are easily washed and migrated into groundwater with the water cycle. Sorption is mainly affected by various environmental factors such as various physicochemical properties of antibiotics, pH values, the content of soil and sediment organic matter, and cation exchange capacity. Sorption is critical for antibiotics entering groundwater [76]. The sorption exchange of antibiotics between water and soil is characterized by the partition coefficient. The results of Boy-Roura et al. [59] showed that the distribution coefficient of organic compounds depends on the soil type and its organic carbon–water distribution coefficient. Antibiotics are complex molecules whose sorption behavior can be determined based on temperature, pH, solid–liquid ratio, ionic strength, initial concentration, and the octanol/water partition coefficient [77]. Kim et al. [78] studied the adsorption of TC, STZ, and amoxicillin in silt and sandy loam soils; the results showed that the adsorption capacity increased with the decrease in soil pH and the increase in organic matter. The adsorption of the three antibiotics in silty loam was significantly higher than that in sandy loam; amoxicillin and STZ were more easily migrated, and TC is easily adsorbed by acidic soils with relatively high organic matter.

### 5.3. Hydrolysis

Hydrolysis is an important environmental behavior for the degradation of antibiotics in the groundwater environment. During the reaction, the parent structure of the antibiotic is broken, and then, one or more degradation products are generated. The hydrolysis process of antibiotics in the groundwater environment is affected and restricted by water environment factors and the physical and chemical properties of antibiotics (such as temperature, pH value, ionic strength, and the solubility of antibiotics, etc.). Through laboratory biodegradability testing with sewage sludge, Längin et al. [79] found that BL is rapidly hydrolyzed, rendering the antibiotic inactive. Loftin et al. [77] explored the factors affecting the hydrolysis of SAs, TCs, and MLs, and the results showed that the ionic strength had no significant effect on the hydrolysis of antibiotics. The hydrolysis rates of CTC, OTC, and TC were significantly affected by pH and temperature, while SAs and MLs had no obvious hydrolysis.

### 5.4. Biodegradation

Although the number of microorganisms in groundwater is very scarce, biodegradation is still an important pathway for the transformation of antibiotics in groundwater. When antibiotic-contaminated water seeps through soil profiles and reaches aquifers, biodegradability is slowed by limited microbial populations, oxygen, and nutrients. The degradation of antibiotics in groundwater is affected by environmental factors, microbial properties, and the molecular structure of antibiotics. For example, SMZ is less biodegradable than other antibiotics in microcosm studies [59]. Maki et al. [80] added ampicillin, DXC, OTC, thiamphenicol, and JOS to fish farm sediments, and found that ampicillin, DXC, OTC, and thiamphenicol were all significantly biodegraded, while JOS was hardly degraded. The study by Martins et al. [81] on the anaerobic biodegradation of antibiotics showed that in the presence of nitrate and sulfate, SMZ exhibited biodegradability, while CIP was removed by 62% and 85%, respectively. The redox environment affects the biodegradation process of antibiotics in groundwater; Gartiser et al. [82] found that doxycycline (DXC) could be effectively removed under aerobic conditions through tank aeration experiments, while ROX, clarithromycin (CAM), and clindamycin (CDM) can be removed under anaerobic conditions.

## 6. ARBs and ARGs in Groundwater

Concentrations of antibiotics are lower in groundwater than in surface water [51], but in the form of persistently low levels in groundwater, microbial resistance is induced in the groundwater and spreads bacterial resistance genes (ARGs) in different bacteria, resulting in highly antibiotic-resistant bacteria (ARBs) that affect human and animal health [83]. ARGs and ARBs proliferate after entering the organism, forming anti-antibiotic modules in the organism [84], reducing the therapeutic effect of antibiotics. Hiller et al. [85] reported that disinfection had little effect on ARGs and ARBs and even induced the development of antibiotic resistance. At present, ARGs have been detected in surface water [86] (such as aquaculture wastewater [31], lakes, and rivers), soil [87], and groundwater [88]. The World Health Organization [26] calls ARBs a global public health crisis that must be managed with the utmost urgency. It is estimated that antibiotic resistance will kill 10 million people globally by 2050, resulting in economic losses of up to 100 trillion US dollars [89]. ARBs in groundwater have been studied in many regions around the world, and a substantial increase in ARBs has been reported in poultry and livestock, especially in low-income countries [90].

This article reviews the emergence of ARGs in the groundwater of China, as shown in Table 5. Li et al. [32] found tetG, tetM, tetO, qnrA, sul1, and sul2 in groundwater in Shunyi, Changping, Daxing, Ninghe, Anping, Guangyang, and Xiqing in China, and the composition and distribution of ARGs in the sewage from the feedlots and the groundwater of nearby villages were similar, indicating that ARGs could infiltrate into the groundwater environment. Zou et al. [91] revealed the occurrence of 18 antibiotic ARGs in the groundwater of the Dabaoshan mine in Guangdong. Gao et al. [83] detected sulfonamide ARGs (sul1 and sul2) in groundwater near a pig farm. Liu at al. [53] also observed sulfonamide ARGs in groundwater near the WWTP. Zhang et al. [92] reported that 75 antibiotic ARGs were found in the groundwater of the Honghu Lake watershed in the middle reaches of the Yangtze River in China. Chen et al. [93] found 171 ARGs in groundwater near a landfill in Xiamen. Wu et al. [94] screened 127 ARGs in the groundwater of the Maozhou River by high-throughput quantitative PCR (HT-qPCR). Huang et al. [95] detected three ARGs on a colivestock farm in Zhongshan City.

## 7. The Ecological Harm of Antibiotics

The ecological harm of antibiotics in the water environment is mainly reflected in two aspects: the impact on aquatic organisms and the disturbance of element circulation in the aquatic ecosystem. There are different types of microorganisms in groundwater. When the residual concentration and time of antibiotics exceed the tolerance limit of microorganisms, they will inhibit the growth of microorganisms or even kill microorganisms, significantly affect the number and types of microorganisms, make the microbial community resistant, disrupt the balance of the ecosystem, and interfere with element circulation.

The impact of antibiotics on the ecosystem is mainly reflected indirectly through the relationship between microbial individuals, populations, and ecosystem functions, including the number, activity, community structure, and toxicity effects of microorganisms [96]. Chen et al. [97] reported that antibiotics can inhibit the growth of microorganisms, resulting in a significant decrease in the number of microorganisms. The results of Schmitt et al. [98] showed that the resistance of the microbial community increased by 10% when the SCP content was 7.3 mg/kg. To study the structural changes of the community, “pollution-induced community tolerance” has been used to evaluate the pollution effects of ecosystems in recent years. The toxicity effect of antibiotics is mainly based on the acute toxicity test of a single strain, and the antibiotic concentration range set in the experiment is usually two orders of magnitude higher than the actual situation. The research on the harm of antibiotics to aquatic animals is mainly in terms of acute toxicity; the results of Zhang et al. [99] showed that tetracycline can significantly inhibit the growth and development of zebrafish embryos, mainly manifested as prolonged hatching, shortened body length, swelling of the yolk sac, and the loss of swim bladders.

The cycle of elements in the water ecosystem usually requires the participation of microorganisms. For example, the processes of biological nitrogen fixation, ammoniation, nitrification, denitrification, anaerobic ammonium oxidation, and nitrate dissimilatory reduction to ammonia involved in the cycle of N in water are inseparable from the action of microorganisms. Both the phosphorus cycle and carbon cycle in water are driven by microorganisms. Since antibiotics have the effect of killing or inhibiting microorganisms, antibiotic residues in the water environment will inevitably affect the circulation of elements. Many studies have confirmed that antibiotics in the water environment can interfere with nitrogen cycling [100] and affect the normal function of phosphorus-solubilizing bacteria [101].

Zhang et al. [17] calculated the potential ecological risk to aquatic organisms of effluent from 12 sewage plants in Dalian City based on risk entropy (RQ), and the results showed that due to the presence of high concentrations of clarithromycin or ERY, more than 40% of effluent samples pose a high ecological risk, and individual antibiotics may present a low to moderate ecological risk. Xing et al. [102] evaluated the ecological risk of antibiotics in rural domestic sewage treatment facilities in the Yangtze River Basin of China, showing that approximately 26.8%, 43.9%, and 14.6% showed high, medium, and low risks, respectively. Therefore, the environmental threat of these antibiotics deserves further attention.

## 8. Prevention and Control of Antibiotic Pollution in Groundwater

The abuse of antibiotics not only pollutes the environment, but also poses a potential threat to human health and the ecology. To alleviate and control the problem of antibiotic pollution, the most important thing is to control the source discharge of antibiotics, and the control and management of antibiotic pollution should also be conducted from the perspective of sewage treatment and groundwater restoration.

As a prominent country in livestock farming and agriculture, China faces greater challenges than other countries in terms of antibiotic pollution. Actions have been taken so far in China, which have regulated the use of antibiotics in animal feed since 1989 [103]. Regulations such as the “2009 Animal-derived Bacterial Resistance Monitoring Plan” (The Ministry of Agriculture Veterinary Bureau Document (2009) No. 010) and the “Notice on Carrying out Special Inspection on the Generation and Disposal of Hazardous Wastes such as Antibiotic Drugs” (The Ministry of Ecology and Environment Document (2009) No. 80) have been promulgated one after another. The Ministry of Agriculture and Rural Affairs of China issued Announcement No. 194 on 10 July 2019, announcing the suspension of the use of some antibiotic feed additives, and resolutely safeguarding food and public health safety. In addition, the potential harm of antibiotics is the generation of resistance genes and the increase in microbial resistance. China has formulated a series of policies for the governance of antibiotic resistance, ranging from drug control policies in the last century, to measures of clinical drug use and regulation in the early 21st century, and then the “‘Healthy China 2030′ Plan Outline” (Documents of the General Office of the Central Committee of the Communist Party of China (2006) NO. 23) and “Water Pollution Prevention and Control Action Plan” (State Council General Office Documents (2015) NO. 17), released in recent years. The government has been active and focused on tackling the problem of antibiotic resistance.

More than half of the antibiotics that have not been fully utilized enter sewage treatment plants in China. Due to the lack of effective treatment technology, some antibiotics will enter the environment. In order to reduce the pollution of antibiotics in the environment, a variety of economically feasible antibiotic wastewater treatment technologies have been developed (Table 6). The activated sludge method and the advanced oxidation method are the main methods for removing organic pollutants in sewage treatment plants at present. The removal of antibiotics and resistance genes by the activated sludge process includes sludge sorption and microbial degradation. Li et al. [104] found that antibiotics in the activated sludge treatment process were mainly removed by the sorption of activated sludge, and the removal rate of tetracyclines was the highest, reaching 92.3~98.0%. Advanced oxidation technology, such as ozone oxidation, Fenton, electrochemical oxidation, and photocatalysis, can effectively remove residual antibiotics in the water environment. Among them, photocatalysis has the advantages of a fast reaction rate, no secondary pollution, and low cost, and is an advanced oxidation technology that has been studied more than the other technologies [105,106,107].

Although measures have been taken at the source discharge of antibiotics and treatment of wastewater containing antibiotics, antibiotics still enter groundwater with improperly discharged sewage, so groundwater contaminated by antibiotics should also be treated. At present, the common remediation technologies for treating pollution in groundwater are divided into ex situ remediation technologies and in situ remediation technologies, according to different technical means [125]. Ectopic restoration technology is a technology that transfers the groundwater contaminated by antibiotics from underground to aboveground by means of pipe and pump extraction, and then treats it in a targeted manner according to the type of antibiotics, which can effectively remove antibiotics. However, it is costly and destroys the original ecological environment. The in situ remediation technology is based on the in situ treatment of antibiotics, without destroying the original soil structure and groundwater natural environment [126], reducing the disturbance to the underground environment and requiring simple handling equipment. Permeable reactive barrier technology (PBR), as a technology for in situ remediation of polluted aquifers, has not been investigated in the research on the filling performance and long-term effect of filling barriers. The technology has been widely used for the removal of inorganics, heavy metals, nitrates, sulfates, and organic pollutants [127,128,129,130,131,132,133].

## 9. Conclusions

This paper reviews the occurrence characteristics of antibiotics in the groundwater systems of major provinces and cities in China in the past decade. The results show that a total of 47 antibiotics in four categories have been detected, and SAs and FQs have the most types and the highest cumulative concentrations.

The problem of antibiotic residues has become a global public health problem. As a major producer and user of antibiotics, China’s antibiotic contamination problem cannot be underestimated. However, at present, there are few reports on antibiotics in groundwater in China, and research on the pollution status and prevention methods of antibiotics is even rarer. Therefore, the research and prevention of antibiotic pollution in groundwater can be carried out from the following aspects. Researchers can develop the most suitable method for the effective, rapid, and low-cost detection of antibiotics to establish a database of antibiotic contamination in groundwater in China, and reveal the migration and transformation rules of antibiotics, which can provide data support for the research and prevention of antibiotic pollution in groundwater in China. Based on the survey results, the government can formulate standards for the content of antibiotics in various media in China, and improve corresponding laws and regulations to manage and strictly control the use of antibiotics. In addition, the contamination of antibiotics in groundwater can be controlled from another important aspect: developing new and effective methods for treating antibiotic-contaminated wastewater.

## Figures and Tables

**Table 1:** TCs and their concentrations detected in groundwater of China (ng/L), and the detection method.

TCs		Beijing	Harbin	Xiantao	Chengdu Shanghai	Bijie	Jianghan Plain			North China Southwest of China
							Shahu County	Four Lakes Basin	Chenhu Lake	
oxytetracycline	OTC	3.2		3.18	39	44.71	152.09	0.10	3.91	
tetracycline	TC	nd		4.77	48	7.51	199.62	0.38	2.63	184.2
chloroetetracycline	CTC	nd		6.16	76	12.19	253.8	0.13	3.71	8
doxycycline	DXC		3.91		39	30.35			5.73	
Detection method		LC–MS/MS, HPLC ESI–MS/MS	UPLC–MS/MS	UPLC–MS/MS	UPLC–MS/MS	UPLC–MS/MS	HPLC–MS/MS	UPLC–MS/MS	LC–MS/MS	UPLC
		[52,53]	[54]	[51]	[45]	[55]	[51]	[56]	[57]	[58]

Note: Blank means no detection, nd means no detection.

**Table 2:** SAs and their concentrations detected in groundwater of China (ng/L), and the detection method.

SAs		Beijing	Harbin	Guilin	Taipei	Xiong’an	Xiantao	Guangxi	Chengdu Shanghai	Bijie	Jianghan Plain			Northern Part of the North China Plain	North China Southwest of China
											Shahu County	Four Lakes Basin	Chenhu Lake		
sulfamethazine	SMZ	10.07	0.78	42.67	28.9	11	1.01		250	2.21	47.1713		0.53		
sulfamerazine	SM1		15.3	7.99		0.17			15			0.31	0.02		
sulfachloropyridazine	SCP			14.71					117					0.44	153.4
sulfamethoxypyridazine	SMP		140.34	14		0.97							0.02		
sulfathiazole	STZ		612			20			32				0.71		
sulfapyridine	SPD		68.6	nd		3.6	0.55	1.47		1.24			0.28		56.4
sulfadiazine	SDZ	17.6	0.43	8.25	14.4	20					27.364	6.26	0.03	45.4	
sulfaquinoxaline	SQX			nd		2.21							1.1		
sulfadimidine	SM2	236					0.5		49	1.03				1.61	3.9
trimethoprim	TMP	8.7		1.35		0.59		1.16	40			0.36		3.18	
sulfadimethoxine	SDM			1.76	4.3	1.67		128							65.5
sulfadoxine	SDX														4.2
sulfamethizole	SMTZ		12.4			0.08									28.7
sulfaphenazolum	SPA		2.61												
sulfametoxydiazine	SMD		4.35												15.6
sulfamethoxazole	SMX	9.41	7.38	16.25	1820	46					50.35	6.28	0.91	11.13	
sulfisoxazole	SIZ								8.4						9.2
sulfamonomethoxine	SMM		1.94			nd	19		29						
sulfacetamide	SA											nd			3.7
Detection method		LC–MS/MS, HPLC ESI–MS/MS	UPLC–MS/MS	UPLC–MS/MS	LC–MS/MS	UPLC–MS/MS	UPLC–MS/MS	UPLC–MS/MS	UPLC–MS/MS	UPLC–MS/MS	HPLC–MS/MS	UPLC–MS/MS	LC–MS/MS	UPLC–MS/MS	UPLC
		[52,53]	[54,63]	[64]	[61]	[65]	[51]	[30]	[45]	[55]	[51]	[56]	[57]	[66]	[58]

Note: Blank means no detection, nd means no detection.

**Table 3:** FQs and their concentrations detected in groundwater of China (ng/L), and the detection method.

FQs		Beijing	Harbin	Taipei	Shijiazhuang	Xiong’an	Changzhou	Xiantao	Chengdu Shanghai	Bijie	Jianghan Plain			Northern Part of the North China Plain	North China Southwest of China
											Shahu County	Four Lakes Basin	Chenhu Lake		
norfloxacin	NOR	4.5	0.89	9.3	4.66	0.18	96.8	23.75	503	8.92	113.38	0.09	11.07	7.92	442
ofloxacin	OFL	13.2	0.05	11.8	2.83	1.6	36.2	0.47	80	16.47	28.077	52.69	7.56		1199.7
ciprofloxacin	CIP	13.3	1.06	nd	4.38	6.38		6.23	155		203.4	0.14	14.83	10.71	100.6
enrofloxacin	ENR	39.4	0.64		5.27	0.24	70.9	7.2	49		427.3	30.16	nd	33.29	48.5
difloxacin	DIF				1.05				35						5.8
sarafloxacin	SAR				0.96	0.29									
oxolinic acid	OXA				1.78										24.6
flumequine	FLU			6.6	4.1	3.01									22.6
pipemidic acid	PPA				1.14										126.4
marbofloxacin	MBF				1.44										
enoxacin	ENO				4.43	7.52									59.5
fleroxacin	FLE				2.98	nd									10.8
lomefloxacin	LOM							2.11	159		123.87		nd		9.1
moxifloxacin	MOX														26.9
nalidixic acid	NDA					7.41									20.5
sparfloxacin	SPA												2.73		13.4
danofloxacin	DAN					0.17									16.9
cinoxacin	CIN														15.4
Detection method		LC–MS/MS, HPLC ESI–MS/MS	UPLC–MS/MS	LC–MS/MS	HPLC–MS/MS	UPLC–MS/MS	UPLC	UPLC–MS/MS	UPLC–MS/MS	UPLC–MS/MS	HPLC–MS/MS	UPLC–MS/MS	LC–MS/MS	UPLC–MS/MS	UPLC
		[52,53]	[54]	[61]	[69,70]	[65]	[71]	[51]	[45]	[55]	[51]	[56]	[57]	[66]	[58]

Note: Blank means no detection, nd means no detection.

**Table 4:** MLs and their concentrations detected in groundwater of China (ng/L), and the detection method.

MLs		Beijing	Harbin	Taipei	Xiong’an	Xiantao	Bijie	Jianghan Plain			North China Southwest of China
								Shahu County	Four Lakes Basin	Chenhu Lake	
roxithromycin	ROX	nd	1.58	nd	nd	0.09	4.95	7.6167	13.90	0.99	54.5
erythromycin	ERY	1.21	23.30	54.80	nd	5.63	1.28	290.81	30.25	12.16	345.7
clarithromycin	CAM			12.50	nd	0.02		0.7605		0.03	
spiramycin	SPI				nd						11.8
josamycin	JOS				nd						16.5
azithromycin	AZM									0.10	
Detection method		LC–MS/MS, HPLC ESI–MS/MS	UPLC–MS/MS	LC–MS/MS	UPLC–MS/MS	HPLC–MS/MS	UPLC–MS/MS	HPLC–MS/MS	UPLC–MS/MS	LC–MS/MS	UPLC
		[52,53]	[54]	[61]	[65]	[51]	[55]	[51]	[56]	[57]	[58]

Note: Blank means no detection, nd means no detection.

**Table 5:** Occurrence of ARGs in groundwater of China.

City	NO. of ARGs Detected	Common	Difference	
Xiamen	171	The vicinity of the sampling site was heavily contaminated with antibiotics, and ARGs were found in the samples	SAs, multidrug and aminoglycosides resistance genes	[93]
Da baoshan min	18		SAs, CPs, and TCs resistance genes	[91]
Shunyi	6		TCs, FQs, and SAs resistance genes	[32]
Changping	6			
Daxing	6			
Ninghe	6			
Anping	6			
Guangyang	6			
Xiqing	6			
Honghu Lake	75		Multidrug and bacitracin resistance genes	[92]
Beijing	9		BLs, TCs, SAs, and MLs resistance genes	[53]
Maozhou	127		SAs, aminoglycosides, MLs, and TCs resistance genes	[94]
Jianzhong, Wentang	19		TCs, FQs, SAs, multidrug, and BLs resistance genes	[83]
Zhongshan	3		TCs and SAs resistance genes	[95]

**Table 6:** Common methods for antibiotics removal.

Technology	
Physical chemical method	Flocculation [108]
	Sorption [109]
	Chemical oxidation [110,111,112,113]: Chlorinated oxidation O_3_ UV Electricity Fenton Fenton-like (PMS/PDS) Combination (UV–O_3,_ O_3_–H_2_O_2_, Fenton–O_3_)
	Membrane separation [114,115]: Microfiltration (MF) Ultrafiltration (UF) Nanofiltration (NF) Reverse osmosis (RO) Electrodialysis (ED)
Biological method	Aerobic biological treatment [116,117,118]: Biological contact oxidation Deep well aeration Biofilter Sequencing batch activated sludge process Biological fluidized bed Membrane bioreactor
	Anaerobic biological treatment [119,120]
Combined process	Flocculation–Electrochemical oxidation–Membrane bioreactor [121]
	Fenton + Activated sludge process [122]
	Bioimmobilization reactor + UV–Fenton [123]
	Advanced oxidation + Membrane bioreactor [124]

## Data Availability

The data presented in this study are available on request from the corresponding author.
